# Isoniazid preventive therapy completion in children under 5 years old who are contacts of tuberculosis cases in Lima, Peru: study protocol for an open-label, cluster-randomized superiority trial

**DOI:** 10.1186/s13063-022-07062-6

**Published:** 2023-01-24

**Authors:** L. Otero, N. Zetola, M. Campos, J. Zunt, A. Bayer, M. Curisinche, T. Ochoa, M. Reyes, V. Vega, P. Van der Stuyft, TR. Sterling

**Affiliations:** 1 Facultad de Medicina, Universidad Peruana Cayetano Heredia, Lima, Peru; 2 Instituto de Medicina Tropical Alexander Von Humboldt, Universidad Peruana Cayetano Heredia, Lima, Peru; 3 Unit of General Epidemiology and Disease Control, Department of Public Health, Institute of Tropical Medicine, Antwerp, Belgium; 4 Division of Pulmonary and Critical Care, Augusta University, Augusta, GA USA; 5 Facultad de Ciencias, Universidad Peruana Cayetano Heredia, Lima, Peru; 6 Department of Neurology, University of Washington School of Medicine, WA Seattle, USA; 7 Facultad de Salud Pública, Universidad Peruana Cayetano Heredia, Lima, Peru; 8 Dirección de Prevención Y Control de Tuberculosis, Ministerio de Salud, Lima, Peru; 9 Centro Nacional de Salud Pública, Instituto Nacional de Salud, Lima, Peru; 10 Department of Public Health, Faculty of Medicine, Ghent University, Ghent, Belgium; 11 Vanderbilt University School of Medicine, Nashville, TN USA

**Keywords:** Tuberculosis, Child, Preschool, Latent *M. tuberculosis*/prevention and control, Peru, Randomized controlled trial

## Abstract

**Background:**

Children < 5 years old in contact with TB cases are at high risk for developing severe and fatal forms of TB. Contact investigation, BCG vaccination, and isoniazid preventive therapy (IPT) are the most effective strategies to prevent TB among children. However, the implementation of IPT faces challenges at several stages of the cascade of care of TB infection among children, particularly those less than 5 years old. In Peru, a large proportion of children do not complete IPT, which highlights the need to design effective interventions that enhance preventive therapy adherence and completion. Although the body of evidence for such interventions has grown, interventions in medium TB incidence settings are lacking. This study aims to test the effectiveness, acceptability, and feasibility of an intervention package to increase information and motivation to complete IPT among children < 5 who have been prescribed IPT.

**Methods:**

An open-label, cluster-randomized superiority trial will be conducted in two districts in South Lima, Peru. Thirty health facilities will be randomized as clusters, 10 to the intervention and 20 to control (standard of care). We aim to recruit 10 children from different households in each cluster. Participants will be caretakers of children aged < 5 years old who initiated IPT. The intervention consists of educational material, and short message services (SMS) reminders and motivators. The primary outcomes will be the proportion of children who picked up > 90% of the 24 weeks of IPT (22 pick-ups) and the proportion of children who picked up the 24 weeks of IPT. The standard of care is a weekly pick-up with monthly check-ups in a health facility. Feasibility and acceptability of the intervention will be assessed through an interview with the caretaker.

**Discussion:**

Unfavorable outcomes of TB in young children, high effectiveness of IPT, and low rates of IPT completion highlight the need to enhance adherence and completion of IPT among children < 5 years old. Testing of a context-adapted intervention is needed to improve IPT completion rates and therefore TB prevention in young children.

**Trial registration:**

ClinicalTrials.gov NCT03881228. Registered on March 19, 2019.

**Supplementary Information:**

The online version contains supplementary material available at 10.1186/s13063-022-07062-6.

## Background

Children exposed to tuberculosis (TB) are at high risk for developing severe and fatal forms of TB, such as TB meningitis, which has a low probability of survival, and a high risk of neurological sequela [1, 2]. In 2020, an estimated 1 million children had TB (11% of the global burden) and 204,000 died (16% of the global burden) [3]. In a recent meta-analysis, the case-fatality ratio for children less than 5 years old was 43.6% [4], compared to 3.0% in adults [5].

To prevent TB in children we need to diagnose and treat adult TB, the main infectious source [6], vaccinate newborns with BCG to decrease the incidence of severe forms of pediatric TB [7], and provide tuberculosis preventive therapy (TPT) to children with latent TB infection (LTBI); the latter reduces TB risk by more than 60% [8–10]. Isoniazid preventive therapy (IPT) is the most widely implemented TPT in high and moderate incidence countries. Other effective regimens such as 3 months of weekly rifapentine plus isoniazid, a 3-month regimen of daily isoniazid plus rifampicin, or 4 months of daily rifampin, are not widely implemented yet. In 2020, 36 countries reported use of rifapentine-containing regimens for research purposes and in some specific populations [9]. IPT delivery faces challenges through the continuum of care, from ruling out active TB to maintaining adherence to a daily dose of isoniazid for 6 months [11]. In 2019, only 29% of household contacts aged less than 5 years received TPT and the number of people initiating TPT dropped from 3.6 to 2.8 million between 2019 and 2020, due to the COVID-19 pandemic [5]. TPT completion rates are not reported by TB programs. However, a systematic review found that approximately 20% of children starting TPT completed it, even in high-income countries[12]. In Peru, a review of national routine surveillance data found that only 22% of eligible children < 5 years old received a full course of IPT between 2015 and 2016 [13], which is consistent with studies in Lima [14, 15].

When evaluating why do children not complete TPT, a scoping review of 146 studies identified concerns about adverse effects, low income, stigma, and transport as barriers and found these factors to be associated with lower retention in care [16]. Conversely, caregiver education and shorter regimens facilitated the completion of TPT [16]. Health system aspects such as poor linkage to TB services, patient’s mistrust of the health system, and lack of commitment to shared responsibility on TPT adherence by providers impacted treatment initiation, adherence, and completion in both adults and children on TPT [12]. A mixed-methods systematic review of 37 studies in high TB incidence settings [17] found that lack of prioritization of pediatric TB prevention, healthcare workers’ knowledge of TB infection, and characteristics of index cases such as female sex and direct kinship with children determined success in completing the full continuum of care.

There is limited evidence on the effectiveness of interventions to improve adherence to and completion of IPT and other TPT, especially in high and moderate TB incidence settings [18, 19]. For every 10 intervention studies in high-income countries (HIC), there is one in low- or middle-income countries (LMIC) [16, 20]. Education, counseling, incentives (monetary and non-monetary) and cultural case management may be effective for IPT completion, but results vary by context and underlying reasons for poor adherence [20, 21]. Directly observed preventive treatment (DOPT) and cash incentives have been implemented, but not tested, to improve TPT completion in children [16]. Daily SMS reminders for TB treatment have not been found to increase adherence [22]. However, weekly SMS reminders have shown promising results for IPT completion and adherence. A community-based intervention to improve adherence to IPT tested in 10 health facilities in Lesotho included mentoring healthcare workers, house visits, IPT-related educational activities, weekly calls, and SMS and showed a higher yield of contacts and treatment completion in the intervention group (RR 1.38 95% CI 1.03–1.84; *p* = 0.03) [23, 24]. A non-randomized study in Lima, Peru of a multi-component intervention with daily SMS reminders showed 72% TPT completion among adult and child contacts [25]. Based on the limited evidence of interventions in medium incidence settings, we will conduct an open-label cluster-randomized superiority trial designed to test the effectiveness and implementation of an intervention package to increase information and motivation to complete IPT among children < 5 who have been prescribed IPT.

## Methods

We adhered to the Standard Protocol Items: Recommendations for Interventional Trials (SPIRIT) 2013 Statement. SPIRIT checklist can be found in Additional file 1: Appendix 1.

### Study setting

The study will be conducted in Villa Maria del Triunfo (VMT) and San Juan de Miraflores (SJM), two districts in South Lima, Peru with a population of 432,835 and 408,538 people, respectively (2019). SJM has twenty-five and VMT twenty-six primary health care facilities under the Ministry of Health with a TB clinic (ESN-PCT, for its acronym in Spanish), providing TB care and prevention services for people living within its jurisdiction. The study will be conducted in the 30 largest of these health facilities, which each have at least one TB case per month,

### Study participants

Eligible participants will be caretakers of children who are (1) < 5 years old, (2) a contact of a TB patient, and (3) have an isoniazid preventive treatment (IPT) prescription from the TB physician. We will exclude caretakers not interested in participating in the study, those who do not have a mobile phone or, since August 2020, a smartphone with internet access using WhatsApp.

### Outcome

The primary outcomes will be the difference in the proportion of children who complete > 90% of the 24 pick-up weeks of IPT (22 pick-ups) and the proportion of children who complete the 24 pick-up weeks of IPT in the intervention and control groups. This will be determined by counting the IPT dispensing dates registered in the back of the TB index case treatment card. A secondary outcome to compare the proportion of children with a urine test positive for isoniazid in a subgroup of both arms was canceled due to the adaptations necessitated by the COVID-19 pandemic.

### Intervention description

We developed the intervention package directed at caretakers and children based on preliminary data in the study population, and behavioral and communication theory. The intervention consists of educational material, SMS reminders, and non-monetary incentives.

#### Rationale for the intervention

The Information-Motivation-Behavioral skills model guided the development of the intervention package [26, 27]. This model hypothesizes that information and motivation increase the likelihood of a behavior goal to occur and also exerts an influence in the acquisition of the behavioral skills needed to conduct and sustain the behavior. Our intervention will target the six constructs of the Transtheoretical Model for behavior change of pre-contemplation, contemplation, preparation, action, maintenance, and termination [28]. We will personalize the risk and reach effective objectives by using narratives from respected persons. We hypothesize that behavior change will occur when the caretaker reads about the particularly high TB risk of a child with household TB exposure and the protective effect of IPT when taken daily for 6 months. We will give cues to action so that the caretaker organizes the pick-up and intake. Through weekly SMS reminders and non-monetary incentives (a children’s storybook; since Aug 2020, a video) we will sustain risk awareness and perception of benefits throughout the 24-week IPT course. A weekly children’s story provides a positive side to balance the fear and stigma that may occur when attending a TB clinic to pick up IPT and places the child as the center of the prevention effort taken by the caretaker. The delivery of the intervention will be done parallelly to the provision of the standard of care which will be further described below.

#### Description of intervention components

##### Educational material

Caretakers in the intervention arm will receive an educational booklet (since August 2020 a video) that delivers information on why the child needs to take IPT, the benefits of taking it, and the risk of not taking it (or taking it intermittently). Practical recommendations to give the medication daily to the child are also provided. We will use an active voice and include behavioral recommendations to use daily reminders such as setting a phone alarm, an explanation about why the recommendations are important, numbers to support the recommendations expressed in familiar terms, and an explanation of the nature of risk [29]. We also included two narratives with a portrait picture, one from a local TB physician and one from a TB nurse, both respected healthcare workers in the study districts. We followed the research-based CDC Clear Communication Index which scores educational materials based on the language used. The caretaker is encouraged to read the booklet and discuss it with family members.

##### SMS reminders

SMS reminders will be sent from a central server a day prior to the scheduled IPT pick-up date for participants in the intervention arm. If a caretaker misses the pick-up, he/she will receive an SMS the next day to encourage resuming IPT. If the caretaker does not come back, we will send up to two more follow-up SMS on days 7 and 14 of the missed visit. If the caretaker goes back after 14 days of having missed a weekly pick-up, we will register the interruption, give the missed chapters to the child, and resume the SMS reminders unless the participant withdraws from the study.

##### Children’s storybook

Caretakers will receive a weekly children’s chapter book or video. The story and pictures were developed by an experienced children’s author and illustrator, and tell the story of a character, his family and where they live.

##### Standard of care (SOC)

The Peruvian standard of care [30] indicates that TB nurses interview recently diagnosed TB cases so they list their household and close contacts, and invite them for a TB screening. Upon presentation to the health facility, the nurse asks about symptoms in adult contacts and requests a smear microscopy if cough is present. All contacts < 14 years old are evaluated by a physician, who may request a chest X-ray, a tuberculin skin test (TST), and smear microscopy if the child can produce sputum. If active TB is a possibility, the child may be referred to a pediatric lung health specialist at the hospital. If active TB is ruled out in the child, the index case has drug-sensitive TB and the child is < 5 years old, the child is prescribed IPT for 6 months. TB diagnostic tests and treatment and IPT are free of cost to patients. Caretakers are verbally counseled to come every week to pick up IPT and to give it daily to the child. The child should be seen once a month at the facility to observe the well-being of the child; if the caretaker observes abdominal pain, vomiting, or jaundice, they should bring the child immediately to the health facility. The staff encourages the caretaker to select an IPT pick-up day that he/she can attend regularly. Adverse events related to isoniazid are rare in children [31–33] and routine monitoring of liver enzymes is not recommended unless the child has malnutrition, preexisting liver disease, or concomitant use of other hepatotoxic drugs [34, 35]. In those cases, the child will be managed by a pediatrician at a referral hospital. Data on contacts are registered on the back of the index case treatment card. The sex, age, kinship, and tests are done to rule out TB are registered for all contacts, and IPT prescriptions and pick-ups are registered for children. There are no structured recommendations or guidance on what to do if a caretaker does not come back to pick up IPT. In practice, the nurse may speak to the index case or conduct a household visit to speak to the caretaker and persuade her/him to resume IPT.

### Study procedures

#### Recruitment

TB nurses will ask eligible participants if they are interested in participating, and those who accept will be approached by the research nurse, who will inform caretakers about the study; those who provide written consent will be enrolled.

#### Randomization

We will randomize 30 clusters — health facilities — 10 to the intervention and 20 to control. We aim to recruit 10 children from different households in each cluster. Twelve of the 48 health facilities were excluded from these classifications as they had less than 1 TB case notification per month. To randomize the 36 health facilities, we classified them into 11 groups: 10 groups of 3 health facilities and one group of six facilities based on the number of TB cases reported in 2018, and the number of children < 5 started on IPT in 2018 as registered in routine health information systems. The group with 6 health facilities had the smallest number of cases per year. To select 2 control facilities and 1 intervention facility, we randomly selected from the group with the highest burden (group 1) until reaching the 10 intervention and 20 control facilities. Only the group classification and not the name of the facility was visible during randomization. Random numbers were generated with the Excel formula Rand() next to each group for the 36 health facilities. A witness set the criteria to the assignment of health facilities from groups 1 to 10. After we completed the assignments, the names of the health facilities were unblinded and the list of intervention and control facilities was made public (Available at bit.ly/3FRTA01). There were three stand-by health facilities for both groups in case a replacement was needed.

Based on 2018 TB case notifications, in which each facility reported an average of 68 TB patients (index cases) per year, and each case has a mean of four household contacts, of which, on average one is < 5 years old and that ~ 10% of children would be non-eligible for IPT (Multidrug-resistant TB contact, contraindications to isoniazid, other) and that 30% of those eligible for IPT would not be started on it (as per our findings), we estimated that more than 150 children would be eligible for the study in 1 year.

#### Data collection plan

**Fig. 1:**
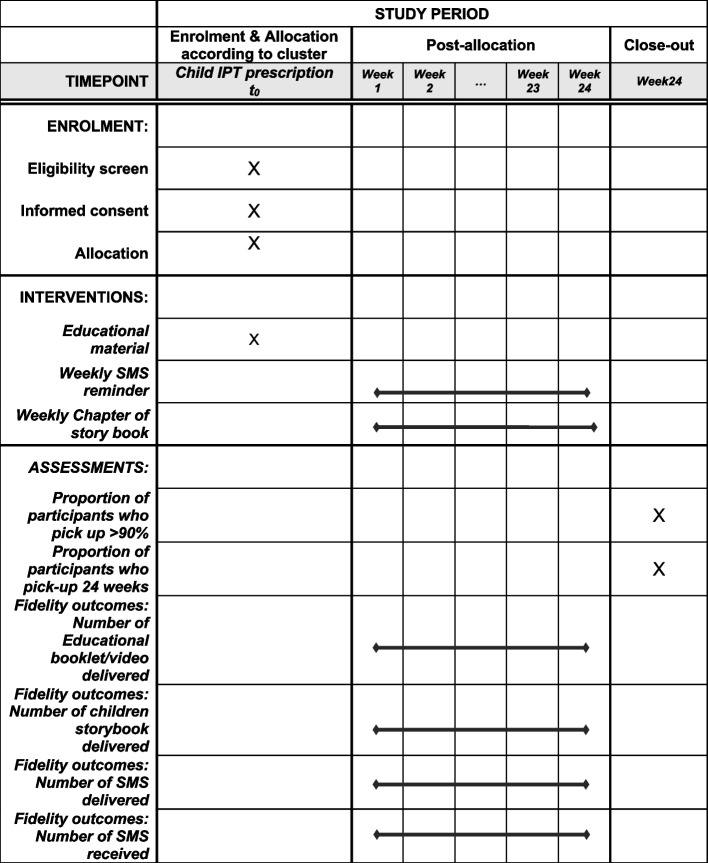
Participant timeline

At enrollment, in the intervention arm, we will collect the following data from the TB treatment card: sex, age, type of TB, smear status, and bacillary load and drug susceptibility test results of the index case. In addition, we will collect age, sex, and kinship to the index case of the child contact. We will also interview the caretaker, to collect the sex, age, occupation, and TB history of the caretaker (if she/he is not the index case), and household socio-economic status and sleeping arrangements of the child and the index case. At the middle and end of the study period, we will review treatment cards to collect data on the main outcome (the dates of IPT pick up) from the back of the TB treatment cards. In the control arm, we will review the TB treatment card to collect index case and child contact characteristics and dates of IPT pick up from the back of the TB treatment cards where it is registered. This will be done at the middle and the end of the study period. A participant timeline is presented in Fig. 1.

#### Power calculations

We expect to increase IPT completion from 30 to 70% with the intervention. A structured behavioral intervention that included self-monitoring and incentives to increase adherence to IPT among children increased IPT completion (OR 2.4, 95% CI 1.7–3.5) [36]. Our intervention will use incentives targeting children and two additional tools targeting the caretaker. Therefore, we expect to increase it by 3.5, the upper CI. In that scenario, the outcome would occur in 70% of the intervention arm. Considering an intra-cluster correlation coefficient of 0.38, we will need to recruit 8 children in each of the 30 clusters (10 intervention, 20 control), to be able to detect that difference with 80% power. Considering potential losses (e.g., children whose IPT is stopped for medical reasons), we will recruit 10 children per cluster. Therefore, we will enroll 100 children in the intervention clusters and 200 children in the control clusters. Sample size calculations were made in Stata v.12 (Stata Corp, 12.0, College Station, TX).

### Data management and analysis

Data will be imported into Stata V16. We will compare the proportion of children who completed IPT in both groups with statistical tests for small groups in an intention-to-treat approach, including all enrolled participants in the denominators. We will also compare the median number of weekly IPT pick-ups. We will compare the characteristics of caretakers, index cases, and children of the intervention and control arm. Missing data will be reported in descriptive tables. Medians and interquartile ranges will be calculated for continuous variables and proportions for categorical variables. Despite randomization, there may be confounding present regarding the probability of completing IPT. Potential confounders include the index case characteristics (sex, age, smear status), child characteristics (sex, age, kinship to the index case), caretaker’s characteristics (sex, age, kinship to the child, and to the index case, occupation, and education) the socio-economic status of the family and the health facility. To determine an unconfounded estimate of the effect of the intervention in the proportion of children who completed IPT, we will test models with a multivariate backward regression analysis. A saturated model with variables associated with IPT completion in our preliminary findings (smear status and treatment outcome of the index case, kinship and TST result of the child [14] and other variables associated (*p* < 0.1) with the outcome in the bivariate analysis will be tested against a model with variables with the weakest associations eliminated one by one. We will analyze the timing of the drop-outs (caretakers not coming back to pick up IPT) with Cox regression and the factors associated to that timing with a multivariate Cox regression analysis. We will report the proportion of enrolled to those eligible overall for the intervention group as well as per health facility, and we will report the full cascade of IPT (evaluation, prescription, initiation, completion) for the intervention and control facilities during the study period. As the data for the outcomes are routinely collected, we will follow the REporting of Studies Conducted using the Observational Routinely Collected Health Data (RECORD) statement to report the study findings [37].

### Fidelity, reach, and feasibility of the intervention

We will register the number of educational booklets and book chapters distributed out of all caretakers in the intervention arm, if more booklets, and chapters than enrolled caretakers have been distributed, we will immediately try to understand to whom and why has it been given as it may be a contamination. If a chapter or video was missed in an IPT pick-up, we will register the occurrence and the reason, and provide two chapters at the next visit. We will record the SMS delivered and the videos and SMS delivered, since August 2021. At the end of the follow-up, we will recruit caretakers from the intervention arm balancing those who completed IPT and those who interrupted or discontinued it and conduct semi-structured interviews. We will explore whether the materials were read, how the content was perceived, how much the materials influenced their daily provision of IPT to the child under their care, the reactions of the children and whether it influenced caretakers, which content worked and why, and what may be missing or unhelpful. We will also explore the effect that the COVID-19 pandemic had on the provision of IPT to the child and if anyone at home developed COVID-19.

### Oversight and monitoring

Daily conduction of the trial is overseen by the principal investigator and the study coordinator. The steering committee is composed of the principal investigator and two co-investigators who meet monthly to discuss progress. The Ethics Committee requests annual reports and can conduct audits at any time. Annual progress reports are submitted to the funder and sponsor.

### Provisions for post-trial care

There is no anticipated harm and compensation for trial participation.

### Limitations and alternative approaches

Contamination may occur if caretakers of different arms share the booklet. Although this is possible, we will recruit participants from a large geographical area of 700,000 population to reduce the clustering of caretakers who know each other. This will be explored with caretakers at the end of the intervention. We will conceal group allocation but cannot blind the intervention to those delivering it or receiving it.

Considering the non-monetary nature of the incentive, we do not expect adverse effects of incentives such as fraudulent practices or the perverse incentive effect in which the incentive provokes the opposite of the intended behavior.

## Discussion

### Adaptations to the COVID-19 pandemic

We report adaptations using the CONSERVE SPIRIT Checklist (Additional file 1: Appendix 2) [38]. On 18 March 2021, approximately 3 months after starting recruitment and because of extenuating circumstances imposed by the COVID-19 pandemic, we stopped in-person recruitment of new participants and the delivery of one of the three components of the intervention, the children’s storybook and continued delivering the SMS to the participants enrolled (*n* = 39). Attendance at primary care facilities was restricted to essential services to reduce the risk of SARS-CoV-2 transmission.

We modified the study protocol and requested an amendment to the UPCH IRB to (1) enroll study participants over the phone after a brief in-person introduction (< 5-min interaction in open air) of the study to reduce contact frequency and duration at health facilities, (2) to deliver the three components of the interventions through WhatsApp (instead of one through SMS and two in physical books), and (3) to include a question in the participant questionnaire, to learn if the participant or somebody at the participant’s house had had a COVID-19 diagnosis, the outcome, and the approximate date of the diagnosis. We developed a 5-min video of the educational booklet with communication specialists and developed 2-min narrated and musicalized videos of the 24 chapters of the children’s storybook so that the three components of the intervention could be delivered by phone. We restarted recruitment in August 2021.

## Trial status

Protocol version 2.0, July 2020. Recruitment began in December 2019. Recruitment was temporarily stopped in March 2020, re-started in August 2020, and is anticipated to end in December 2022. Follow-up will continue until June 2023.

## Dissemination policy

We will disseminate our results as peer-reviewed manuscripts, oral presentations or abstracts. Technical briefs will be prepared to disseminate relevant findings to the National TB Program and participant health facility.

## Supplementary Information

**Additional file 1: Appendix 1.** SPIRIT 2013 Checklist. **Appendix 2.** CONSERVE-SPIRIT Checklist.

## Data Availability

All data generated or analyzed during this study will be made available on request upon completion of the study.

## Abbreviations

TB: Tuberculosis
BCG: Bacille Calmette-Guérin
TPT: TB preventive therapy
SMS: Short message services
IPT: Isoniazid preventive therapy
LTBI: Latent TB infection
COVID-19: Coronavirus disease of 2019
LMIC: Low- or middle-income countries
HIC: High-income countries
DOPT: Directly observed preventive treatment
SPIRIT: Standard Protocol Items: Recommendations for Interventional Trials
VMT: Villa Maria del Triunfo
SJM: San Juan de Miraflores
ESN-PCT: Estrategia Sanitaria Nacional de Prevención y Control de la Tuberculosis
CDC: Centers for Disease Control and Prevention
SOC: Standard of care
TST: TB preventive therapy
SARS-CoV-2: Severe acute respiratory syndrome coronavirus 2
SIDISI: Sistema Descentralizado de Información y Seguimiento a la Investigación
